# The impact of community-based prevention on quality of life—The necessity to control for general health trends the Northern Sweden MONICA study in 2014

**DOI:** 10.1371/journal.pone.0256872

**Published:** 2021-09-02

**Authors:** Elin Spege, Kristina Ek, Stefan Söderberg, Mats Eliasson

**Affiliations:** 1 Department of Business Administration, Technology and Social Sciences, Economics Unit, Luleå University of Technology, Luleå, Sweden; 2 Department of Public Health and Clinical Medicine, Section of Medicine, Umeå University, Umeå, Sweden; 3 Department of Public Health and Clinical Medicine, Sunderby Research Unit, Umeå University, Umeå, Sweden; Sciensano, BELGIUM

## Abstract

**Background:**

The Västerbotten intervention program (VIP), is a public health promotion program in northern Sweden with the aim of preventing cardiovascular disease. Positive effects have been reported although the evidence is not unequivocal. Since only historical controls have been used, effects from other sources than the program have largely been uncontrolled for and health related quality of life (HRQoL) has not been evaluated.

**Purpose:**

By using the neighbouring county of Norrbotten (NB) as the reference population, we compare HRQoL in Västerbotten (VB) and in NB.

**Methods:**

In 2014 the Northern Sweden survey, Monitoring of Trends and Determinants in Cardiovascular Disease (MONICA), examined a random sample from the two counties. HRQoL was measured with the EQ-5D-3L. In total, 1112 subjects aged 40–74 years participated, 516 in VB and 594 in NB. Differences in mean QoL between VB and NB were analysed via Student’s t-test and the Pearson chi-square test.

**Results:**

Average HRQoL measured by the EQ-5D-index was 0.798 in VB and 0.811 in NB, a difference of 0.013 (p = 0.2, CI -0.009 to 0.036). For subjects aged 45–54 years, the HRQoL was lower in VB than in NB, a difference of 0.048 (*p* = 0.041; CI 0.002 to 0.0094). Men had higher HRQoL than women, and university educated had higher HRQoL than those without university education. EQ-VAS showed similar results. Subjects from NB and from VB did not differ regarding age, gender and level of education. In NB, HRQoL decrease with age, a pattern not seen in VB.

**Conclusions:**

We found similar levels of HRQoL in VB and in NB.

## Background

Health problems due to lifestyle such as e.g. obesity, unhealthy diets, lack of physical activity and use of tobacco and alcohol are major causes of premature deaths in all high-income countries [1]. In the mid 1980s, the most northern counties in Sweden, Norrbotten (NB) and Västerbotten (VB), reported the highest mortality rates from cardiovascular diseases in the country. In 1986 mortality rates were about 10 percent higher in NB and VB than in the country as a whole. A community based primary care intervention program, the Västerbotten Intervention Program (VIP), was therefore launched in VB, with the aim of reducing morbidity and mortality from cardiovascular diseases and diabetes [2]. No similar program has been implemented in NB.

While a lifestyle program in the same region targeting high-risk individuals has been shown to reduce cardiovascular risk [3], and demand for societal and health resources [4], the impact of population based public health promotion programs are not trivial to assess. Studies evaluating the effects of the VIP specifically are inconclusive; a report from 2015 draws relatively strong conclusions regarding reduced mortality from cardiovascular diseases [5] while a report from 2019 concludes that there is no evidence that VIP has contributed to any additional reduction of morbidity and mortality from cardiovascular disease when comparing trends with the three neighbouring counties [6]. Still, several studies have evaluated the effects of the VIP program on intermediate outcomes and report improvement on lifestyle parameters such as reducing smoking [7], increasing physical activity [8] and that self-reported health has improved [9], in particular in those with initial poor and fair health. A study of time trends in cardiovascular risk factors in VB, using the MONICA Study, showed a faster improvement in blood pressure and smoking but no difference in the prevalence of diabetes, cholesterol levels or BMI compared to the neighbouring county of NB [10].

A methodological limitation with many of these evaluations is that they lack use of reference group. It is therefore not possible to evaluate whether any effects are a result of the intervention program or stemming from other sources that are not controlled for such as secular trends in the general Swedish society, not specific for VB.

The purpose of this paper is to evaluate if there is a difference in HRQoL between NB and VB. The two regions share similar demographic and socio-economic structure and have developed in parallel for many years. Our hypothesis is that 20 years of the VIP should have had a health-promoting and disease-preventing effect in VB, leading to higher HRQoL of the population of VB than in NB. By using the 2014 Northern Sweden MONICA population-based survey performed in both VB and the neighbouring county of NB, where no such program has been implemented, we are able to control for general health trends by using NB as the reference population.

## Methods

### Study setting

Both NB and VB share similar socioeconomic and demographic characteristics, and the educational levels of the populations have been increasing at similar rates during the last decades. The cities in both regions are mainly located along the coast, with one major university city in each county, while the hinterlands are sparsely populated and have been suffering from shrinking and ageing populations since the 1960s [11].

The VIP is based in primary health care and focuses on health promotion by counselling and support for life style change with the aim of reducing morbidity and mortality from cardiovascular diseases and diabetes. The program has been described in full in previous studies [2]. Since 1995, all individuals of age 40, 50 or 60, living in VB, are invited to a health examination where e.g. body mass index, blood pressure, glucose tolerance, and blood lipids are measured. The health examination also includes a comprehensive questionnaire covering lifestyle, socioeconomic- and psychosocial conditions and general health condition. The program also includes individual advice by a nurse. Annually, about 6000–7000 health examinations, relatively evenly distributed over the age groups, are carried out in VB. The participation rate has varied, with an average of 63 percent participating over the period of 1990–2017 [12]. No similar program has been implemented in NB.

### Data

The World Health Organisation (WHO) MONICA Study was initiated as a multinational project with the purpose of monitoring trends and determinants in cardiovascular disease, it includes repeated population-based surveys but no interventions. The Northern Sweden MONICA Study, which has been described in depth elsewhere [13], consists of the northernmost counties in Sweden, VB and NB.

The present study was approved by the Regional Ethical Committee at Umeå University (Umeå, Sweden 2013, dnr 2013/97-21). In brief, data is collected every 4–5 years by screening a subsample of the general population regarding health status, including cardiovascular disease risk factors. Self-reported socio-demographic information about the participants marital status, household composition, place of residence and country of birth, is also collected through questionnaires. In the 2014 MONICA Study [14] the subjects were asked to rate their quality of life. The invited subjects were randomly selected yet stratified for gender into 10-year age groups (25–34, 35–44, 45–54, 55–64, and 65–74). At each data-collection point, a total of 250 men and 250 women from the counties, in each 10-year age group, were invited, a total of 2500 invited subjects. Since the participants of the VIP are 40 years or older, the MONICA data used here is restricted with 40 years as the lower cut-off.

Results from telephone interviews with the majority of non-participants in earlier surveys did not indicate significant differences compared to participants; non-participants had somewhat lower BMI, and were more prone to smoke, but had similar levels of educational attainment, compared to participants [15]. To investigate the generalizability of the MONICA sample 2014 we compared the VB and NB samples to their regional population averages with respect to age, gender, and education. Results are presented in the following sections. The regional population is defined as the inhabitants residing within the geographical region of VB and NB, year 2014.

### Measurements

#### Quality of life

The standardized questionnaire developed by the EuroQol Group, the EuroQol 5 dimension (EQ-5D), was used to measure the health-related quality of life (HRQoL) [16]. The three level version (EQ-5D-3L), consists of two parts. Part one includes five questions related to each of the five dimensions of health: mobility, self-care, usual activities, pain/discomfort and anxiety/depression. Each dimension has 3 levels: no problems = 1, some problems = 2, and extreme problems/unable to = 3. The subject is asked to indicate health status by choosing the most appropriate level for each of the five dimensions. The digits for the five dimensions (ranging from 1–3) are combined into a 5-digit number that describes health state, ranging from 11111 to 33333. The health states are converted into a single index value (EQ5D-index), ranging between 0 (death) and 1 (full health), by using the same procedure as for the UK tariff value set [17]. For health states perceived worse than death negative index values can be generated.

Part two of the EQ-5D-3L questionnaire consists of a vertical visual analogue scale (EQ-VAS), ranging between 0 and 100, where 0 represents the worst imaginable health state and 100 represents the best imaginable health state. The participant is asked to indicate health status by drawing a line on the scale. The index value (ranging from 0 and 1) and the VAS-measure can be used as a quantitative measure of HRQoL. The EQ-5D-3L is a widely used instrument for measuring HRQoL and has been shown to have good reliability and validity in the Swedish context [18].

#### Education

In the MONICA data, university education is defined as completing an education given at a college or university. In the National Statistics, university education is defined as post-secondary education 3 years or more, that is, a bachelor degree or higher.

#### Characteristics of the sample

In the age group 40–74, 1110 of the 1750 invited participated, 516 from VB, and 594 from NB, which corresponds to a participation rate of 63 percent. In the MONICA sample, 35% of all subjects from VB, and 30% from NB, have a university education (Table 1), which is higher than in their respective regional population [11]. In the MONICA sample, 49% were women in VB and 53% in NB, which is similar to the regional populations. The average age was similar in the counties, both in the MONICA sample and in the regional populations.

**Table 1:** Sample characteristics in comparison with the regional population.

		Participants MONICA Study	Regional population age group:40–74
	County	Mean	Mean
University education (%)	VB	35	19
	NB	30	16
Proportion women (%)	VB	49	50
	NB	53	49
Average age	VB	57	57
	NB	57	57

### Statistical methods

Differences in mean HRQoL between VB and NB were analysed via Student’s t-test. Potential differences between VB and NB within the five dimensions of health was analysed via the Pearson chi-square test. Determinants of HRQoL were analysed in a multivariable linear regression model including age, gender and academic degree as explanatory variables. To test if HRQoL differed between the counties, we performed two multivariable linear regression models with HRQoL as dependent variable including an interaction term for VB county and age, one model with the EQ-5D-index and one with the EQ-VAS. The one-way-ANOVA-test, and the welch-test, showed that pooling the two subsamples is appropriate. Results are given with 95% confidence intervals (CI).

## Results

### Health related quality of life–EQ-5D-index and EQ-VAS

Subjects from VB and NB report a mean EQ-5D-index and EQ-VAS of approximately 0.8, with no significant differences between the counties (Table 2).

**Table 2:** HRQoL by EQ-5D-index and EQ-VAS stratified by gender and county.

	County	Mean	Std. Deviation	P-value difference of means; CI
**EQ-5D-index**				
Average	VB	0.80	0.19	0.2; -0.04 to 0.01
	NB	0.81	0.19	
Men	VB	0.82	0.17	0.1; -0.05 to 0.06
	NB	0.85	0.16	
Women	VB	0.77	0.21	0.6; -0.04 to 0.02
	NB	0.78	0.21	
**EQ-VAS**				
Average	VB	0.79	0.16	0.2; -0.03 to 0.01
	NB	0.80	0.17	
Men	VB	0.81	0.15	0.1; -0.05 to 0.01
	NB	0.83	0.15	
Women	VB	0.76	0.17	0.6; -0.04 to 0.05
	NB	0.77	0.17	

The difference in EQ-5D-index between the counties was 0.01 (p = 0.2; CI -0.04 to 0.01) and the difference in EQ-VAS between the counties is 0.01 (p = 0.2; CI -0.03 to 0.01). While women in both counties rate their QoL lower than men, no significant differences between women from VB and NB, nor between men in the two counties with respect to EQ-5D nor EQ-VAS were found.

### Differences within the HRQoL dimensions

Table 3 presents the responses for the 3 levels of each dimension for each county. Both in VB and NB, participants reported more problems on the dimensions of pain/discomfort and anxiety/depression than on the other dimensions. Only small proportions, primarily in the pain/discomfort dimension, reported extreme problems. Similar proportions in NB and VB (33% respectively 32%) reported “no problems” on all the five dimensions. No significant differences between the counties in any of the dimensions were found, p-values ranging between 0.1 to 0.4.

**Table 3:** Proportion respondents reporting moderate or severe problems on each EQ-5D dimension; %.

*Mobility*	NB	VB
Moderate problems (2)	10	8
Severe problems (3)	0	0
*Self care*		
Moderate problems (2)	1	3
Severe problems (3)	0.4	0
*Usual activities*		
Moderate problems (2)	7	7
Severe problems (3)	0	0
*Pain/discomfort*		
Moderate problems (2)	54	55
Severe problems (3)	4	4
*Anxiety/depression*		
Moderate problems (2)	29	32
Severe problems (3)	1	2

### Differences within age groups across counties

Table 4 presents the self-reported HRQoL and EQ-VAS for different age groups for the two counties.

**Table 4:** EQ-5D-index and EQ-VAS, by age group and county.

EQ-5D-index Age Group	County	n	Mean	Std. Deviation	P-value difference of means; CI
40–44	VB	70	0.81	0.19	0.1; -0.10 to 0.01
	NB	65	0.85	0.12	
45–54	VB	143	0.78	0.23	0.04; -0.09 to-0.00
	NB	176	0.83	0.17	
55–64	VB	151	0.80	0.19	0.7; -0.04 to 0.05
	NB	189	0.79	0.22	
65–74	VB	152	0.81	0.15	0.7; -0.03 to 0.05
	NB	164	0.80	0.20	
**EQ-VAS**	County				
Age Group					
40–44	VB	70	0.79	0.16	0.2; -0.08 to 0.02
	NB	65	0.83	0.13	
45–54	VB	143	0.78	0.18	0.08; -0.07 to-0.00
	NB	176	0.81	0.15	
55–64	VB	151	0.78	0.16	0.2; -0.03 to 0.04
	NB	189	0.78	0.18	
65–74	VB	152	0.79	0.14	1.0; -0.04 to 0.03
	NB	164	0.79	0.17	

The EQ-5D-index in the age group 45 to 54 years is slightly lower in VB than in NB. No such pattern was found for the EQ-VAS. The results from the two multivariable linear regression models, including also an interaction term for VB county and age, one with the EQ-5D-index and one with EQ-VAS, are presented in Table 5.

**Table 5:** Multivariable linear regression model with interaction term between Västerbotten county and age.

						95% Confidence Interval	
Dependent variable	Independent variables	B	Std. Error	Sig.	t	Lower bound	Upper bound
**EQ-5D-index**	Intercept	0.915	0.048	0.000	18.995	0.820	1.009
	No univ.educ	-0.055	0.012	0.000	-4.487	-0.079	-0.031
	VB	-0.161	0.069	0.020	-2.336	-0.296	-0.026
	Men	0.064	0.011	0.000	5.569	0.041	0.086
	Age	-0.002	0.001	0.045	-2.010	-0.003	0.00
	VB*age	0.003	0.001	0.035	2.113	0.000	0.005
**EQ-VAS**	Intercept	0.882	0.041	0.000	21.382	0.801	0.962
	No univ.educ	-0.047	0.011	0.000	-4.419	-0.067	-0.026
	VB	-0.114	0.059	0.054	-1.931	-0.230	0.002
	Men	0.056	0.010	0.000	5.734	0.037	0.075
	Age	-0.001	0.001	0.056	-1.915	-0.003	0.000
	VB*age	0.002	0.001	0.091	1.689	0.000	0.004

The one-way-ANOVA-test shows that the variance related to HRQoL and age differs between the counties. The multivariable linear regression model (Table 4) shows that for the EQ-5D-index the interaction term between age and county is implying that the effect of age on HRQoL differs between the counties. No such effect is found for EQ-VAS.

## Discussion

### Results

The purpose of this paper was to evaluate if there are any difference in HRQoL between the populations in VB and NB, given that VB has had a community-based health-promoting program for over 20 years. Both NB and VB share similar socioeconomic and demographic characteristics, as well as a history of having the highest reported mortality rates from cardiovascular diseases in Sweden. The MONICA data, collected in VB and NB, gives a unique opportunity to use the subjects from NB as a reference population. In addition, The MONICA data gives the possibility to study potential differences in HRQoL between the counties from a societal perspective, rather than its effect on the individuals in the intervention group. The effect of public health promotion programs on population based public health, which is the purpose of the VIP, depends not only on the effectiveness of the health promoting activity, but also on the ability of the program to reach the citizens and achieve long-lasting changes in life-style.

With respect to gender and age, there are no large differences between the participants from VB and NB, nor in comparison with the regional averages as reported by Statistics Sweden (11). Approximately one third of the participants from both VB and NB report that they have a university degree, which is a substantially higher proportion than in the regional populations. The HRQoL levels in both NB and VB in the 2014 MONICA survey are higher than what was found in a recent national Swedish study. Burström et al. [19], report average EQ-VAS score on a national basis to be 0.76 while it was 0.79 (VB) and 0.80 (NB) in the MONICA data. These differences can to some extent be a result of different age groups; Burström et al. included ages above 95 years while the results presented here are restricted to participants between 40–85. The higher HRQoL in the 2014 MONICA data may also be a result of sample selection; education level is positively related to HRQoL [20] and participants with higher education seem to be over represented in the sample. It is possible that the differences between the sample and the regional populations to some extent stem from differences in how education is defined in the MONICA survey and national statistics; while the MONICA survey defines university education as completing an education given at a college or university, the national statistics includes Bachelor’s degrees and higher. Since the difference in educational attainment in comparison to the regional averages are similar for both counties, and we are interested in any difference between counties, the problems caused by selection bias have likely not affected the results substantially.

We found no significant differences in health-related HRQoL between the population of VB, with the health intervention program, and NB, without any similar program. Previous research where VB is compared to another county, also based on the 2014 MONICA population survey and using similar methods as in this report, reported a faster decline in blood pressure and smoking in VB but similar trends for diabetes, cholesterol and obesity [10]. The results presented here are different from a previous evaluation of the impact of the program on self-reported health, in which a significant and positive effect was reported [9]. One possible explanation for these contradicting results is that the previous evaluation was based on the outcomes before and after the program was implemented, and did not compare the outcome with any reference population; they did not control for other factors that may have affected HRQoL over time. It should also be noted that self-reported health is not the equivalent of HRQoL.

Results from the multivariate linear regression models also show that men and university educated, in both counties, report higher HRQoL than women and those without university education. These results are consistent with findings in the previous literature [21, 22].

Even though we do not find any evidence of differences in global HRQoL as measured by EQ-5D, it is possible to expect some of the EQ-5D dimensions to be more sensitive to changes in lifestyle. Positive effects of physical exercise on anxiety and depression, as well as on pain are well documented in the literature [23, 24], and since the VIP promotes physical exercise, a higher share of “no problems” reported in the VB sample could be expected within these dimensions if the program has succeeded to increase physical activity in the VB population. However, no differences within any of the EQ-5D dimensions between the two counties were found.

It could be reasonable to expect the effect of the program on HRQoL to be more visible for older participants since the aim of the VIP is to prevent future morbidity and mortality from cardiovascular diseases and diabetes. Also, since the VIP invites all individuals aged 40–60 in VB every 10 years, older citizens have had the chance to be exposed to the program during more than once, which may increase the probability of improving lifestyle. We do however only find a difference for the EQ-5D-index between the counties in one of four age groups; younger participants in the NB have a higher HRQoL than the participants of similar age in VB. Thus, we do not find evidence that global HRQoL is higher in VB, neither within any EQ-5D dimension nor within any age group.

Still, although the results do not support that the VIP has improved HRQoL, results show that although younger participants from VB have a lower HRQoL, HRQoL is not lower for participants of higher age in the VB sample as it is in the NB sample. Based on the findings in previous studies, in Sweden and elsewhere, health status has generally been found to decrease with age [25, 26].

### Policy implications

To break the trend with increasing lifestyle related morbidity and mortality, evidence-based population based preventative measures are needed. To make resource-efficient decisions, policy makers need information about the outcome as well as the costs of measures available. The main contribution of this study lies in pointing at the importance of controlling for changes in HRQoL stemming from other sources than the program; i.e. by comparing the population of the intervention area with a similar reference group. However, for broad population-based programs suitable reference groups are often not available [27]. In such cases it is important for policy makers to be aware of the validity issues stemming from not controlling for possible changes over time in general health or lifestyle.

### Limitations

One limitation with this study is that we only have one point of measure for the HRQoL. If data over HRQoL from multiple points over time had been available, it had been possible to account for any starting point differences between the intervention- and control group. Future MONICA surveys will collect such data and this is an important avenue for future research. Another limitation is that we are unable to control for possible differences in the share of individuals in the MONICA data having taken part in the VIP and the share of VIP participants in the population of VB at large. The participation rate of the 2014 MONICA Study was 63 percent and similar in the two counties, and although this is not significantly different from previous years, a relatively high share of non-participants constitutes a risk of selection bias. Nevertheless, the subsample from the general population invited to participate in the MONICA Study is randomly selected, and has been shown to adequately represent the general population with regard to sociodemographic aspects [15].

Also, although the EQ-5D-3L is found to perform well for many health conditions, there are studies who have questioned the instrument’s ability to accurately capture the HRQoL of the general population [28]. According to the findings of these studies, the ability of EQ-5D to capture HRQoL varies by dimension. The domains of self-care and usual activities did not help explaining the HRQoL significantly in the general population. Adding more dimensions (such as concentration, sleep, and sexual activity) have however only resulted in small improvements in explaining individual differences in HRQoL. The overall conclusion in the literature is that the widely used EQ-5DL-3L is a valid instrument for measuring HRQoL in the general population. Another limitation of the study is the potential of spillover effects of the VIP on to the population of NB (and the rest of the country). Finally, the risk of spurious result due to mass comparisons should be noted, *i*.*e*. the difference found in a single age group within eight comparisons.

## Conclusion

We do not find any difference in HRQoL between VB and its neighboring county NB, despite that the population in VB have been exposed to a public health promotion program for over 20 years.
